# Discovery of a novel *Keithomyces* species isolated from the sclerotia and a new *Papiliomyces* from Southwest China: Integrated taxonomy based on morphology and phylogeny

**DOI:** 10.3897/mycokeys.136.190405

**Published:** 2026-07-15

**Authors:** Dexiang Tang, Hui Chen, Ou Huang, Ling Tao, Qi-Rui Li, Yao Wang, Xiangchun Sheng

**Affiliations:** 1 State Key Laboratory of Discovery and Utilization of Functional Components in Traditional Chinese Medicine & School of Pharmaceutical Sciences, Guizhou Medical University, Guian New District, Guizhou 561113, China State Key Laboratory of Discovery and Utilization of Functional Components in Traditional Chinese Medicine & School of Pharmaceutical Sciences, Guizhou Medical University Guizhou China; 2 The High Efficacy Application of Natural Medicinal Resources Engineering Center of Guizhou Province, Guizhou Medical University, Guian New District, Guizhou 561113, China The High Efficacy Application of Natural Medicinal Resources Engineering Center of Guizhou Province, Guizhou Medical University Guizhou China

**Keywords:** Clavicipitaceae, entomopathogenic fungi, new species, taxonomy

## Abstract

Two new Clavicipitaceae species, *Papiliomyces huangpingensis***sp. nov**. and *Keithomyces ophiocordycipiticola***sp. nov**., were described based on integrated morphological features and multi-locus phylogenetic analyses. The studied materials were collected from Guizhou and Yunnan provinces, China. Phylogenetic analyses based on six loci (nrSSU, ITS, nrLSU, *tef-1α*, *rpb1*, and *rpb2*) strongly supported the monophyly of the genera *Papiliomyces* and *Keithomyces*, with *P. huangpingensis* resolved as sister to “*P. sinensis*” and forming a well-supported independent phylogenetic lineage. Morphologically, *P. huangpingensis* resembles *P. albostromaticus* and “*P. sinensis”* in possessing fleshy, clavate, gray stromata and a *Paecilomyces*-like asexual morph, but is clearly distinguished by its markedly shorter and stouter stromata and the presence of two distinct conidial morphotypes. Phylogenetic analyses placed *Keithomyces ophiocordycipiticola* as a sister species to *K. echinosporus* with strong statistical support, from which it can be distinguished morphologically by its significantly shorter conidiophores and occasionally serrate conidia. In addition, *K. ophiocordycipiticola* was isolated from the sclerotia of *Ophiocordyceps* sp., representing an unusual ecological occurrence within the genus. These findings enrich the species diversity of *Papiliomyces* and *Keithomyces* and contribute to a better understanding of species delimitation, host association, and evolutionary relationships within Clavicipitaceae.

## Introduction

Fungi represent one of the most diverse and functionally important groups of eukaryotic organisms, playing essential roles in ecosystem functioning and exerting profound influences on human activities (Mueller and Schmit 2007). With approximately 2,000 fungal species described annually, current knowledge still accounts for only a fraction of the estimated global fungal diversity (Cheek et al. 2020). Entomopathogenic fungi have attracted increasing attention due to their ecological significance and potential applications in biological control, agriculture, and medicine (Yang et al. 2021). Nevertheless, the taxonomy and diversity of this group remain incompletely understood, and many species await discovery and formal description.

Cordycipitoid fungi, traditionally treated under *Cordycepssensu lato* (*s. l*.), constitute one of the most representative lineages of entomopathogenic fungi within the order Hypocreales. Members of this group are typically characterized by filiform ascospores that often disarticulate into secondary ascospores, cylindrical asci with thickened apices, and well-developed stromata (Mains 1958; Kobayasi 1982; Rossman et al. 1999; Hywel-Jones 2002; Sung et al. 2007a). Advances in multilocus phylogenetic analyses have profoundly reshaped the traditional morphology-based classification of *Cordyceps* s. l., resulting in its redistribution among four families, namely Clavicipitaceae, Cordycipitaceae, Ophiocordycipitaceae, and Polycephalomycetaceae (Chaverri et al. 2005; Sung et al. 2007a; Zare and Gams 2007; Johnson et al. 2009; Kepler et al. 2012a, 2012b, 2014; Quandt et al. 2014; Spatafora et al. 2015; Tsang et al. 2016; Mongkolsamrit et al. 2019; Xiao et al. 2023). These revisions have significantly improved the naturalness and stability of higher-level classifications within Hypocreales.

Within this revised framework, the taxonomy of *Metarhizium* and its allied genera has undergone extensive re-evaluation. *Metarhizium* is a widely distributed genus of entomopathogenic fungi, traditionally circumscribed based on anamorphic morphological traits (Tulloch 1976; Driver et al. 2000; Kepler et al. 2014; Brunner-Mendoza et al. 2019). However, morphological convergence and the presence of cryptic species have long impeded accurate species delimitation. The integration of molecular data and multilocus phylogenetic analyses has revealed that the traditional concept of *Metarhizium* comprises multiple phylogenetically distinct lineages, prompting the establishment of several segregate genera (Johannes et al. 2011; Rehner et al. 2011; Huzefa et al. 2017).

As a result, Mongkolsamrit et al. (2020) revised *Metarhizium* and introduced several new genera, including *Papiliomyces*, *Purpureomyces*, and *Keithomyces*. *Papiliomyces* was established to accommodate species previously placed in *Metacordyceps* and *Metarhizium*, and is characterized by solitary to multiple stromata ranging from robust to wiry, superficial to completely immersed perithecia, cylindrical asci, and ascospores that remain entire with septation or disarticulate into cylindrical part-spores (Kepler et al. 2012a, 2012b; Mongkolsamrit et al. 2020). Although the type species, *Papiliomyces liangshanensis*, was later transferred to *Ophiocordyceps* based on morphological and phylogenetic evidence (Wang et al. 2021), the genus *Papiliomyces* remains a distinct lineage within Clavicipitaceae, with its taxonomic boundaries still requiring clarification through additional species-level studies.

*Keithomyces* was also erected by Mongkolsamrit et al. (2020) as a phylogenetically distinct genus closely related to *Metarhizium* and *Purpureomyces*. Species of *Keithomyces* are primarily isolated from soil and exhibit *Paecilomyces*-like asexual morphs, characterized by conidiophores bearing divergent whorls of two to four phialides and echinulate to aciculate conidia. Compared with *Papiliomyces* and typical entomopathogenic species of *Metarhizium*, *Keithomyces* displays distinctive morphological features and has been reported from different ecological substrates, yet its species’ diversity remains poorly explored.

Southwestern China has been recognized as an important hotspot for the diversity of entomopathogenic fungi, although systematic surveys of many cordycipitoid lineages remain limited (Shrestha et al. 2016). During ongoing investigations of cordycipitoid fungi in this region, we collected two fungal specimens that are morphologically and phylogenetically distinct from all known species. Based on detailed morphological comparisons and multilocus phylogenetic analyses, one of the new taxa is assigned to *Papiliomyces*, while the other represents a new species of *Keithomyces*. The present study provides formal descriptions of these two new species and contributes to a better understanding of species diversity and phylogenetic relationships within Clavicipitaceae.

## Materials and methods

### Specimen collection and isolation

Most of the specimens examined in this study were collected from Guizhou and Yunnan Province, China. During field surveys, specimens were photographed in situ and relevant ecological data were recorded. After collection, samples were temporarily stored at low temperatures (approximately 4 °C) in sealed plastic containers and transported to the laboratory for further study. All specimens and dry cultures have been deposited in the Guizhou Medical University Herbarium (GMB). In the laboratory, samples were surface-disinfected by soaking in 30% hydrogen peroxide for five minutes, followed by two rinses with sterile distilled water. Excess moisture was removed using sterile filter paper (Sun et al. 2022). The outer fungal tissue was aseptically excised and transferred onto potato dextrose agar (PDA) plates. Purified cultures were incubated at 25 °C and subsequently maintained on PDA slants at 4 °C for preservation (Wang et al. 2023). Living cultures were stored at the Guizhou Medical University Culture Collection (GMBC).

### Morphological observations

Macromorphological features, including the color, shape, and length of the colony and specimen, were examined and photographed using a Canon 750D camera (Canon Inc., Tokyo, Japan). The asexual morph was observed from cultures grown on potato dextrose agar (PDA). Diagnostic characters of the asexual morph, including the morphology of conidiophores, phialides, and conidia, were examined. Anamorphic structures were mounted in lactophenol cotton blue for detailed microscopic observation under a Nikon ECLIPSE Ni compound microscope (Nikon, Japan) equipped with a Canon EOS 700D digital camera. Microscopic observations and photomicrographs were obtained, and measurements were made using Tarosoft Image Framework (v. 0.9.7).

### DNA extraction, PCR amplification and sequencing

Genomic DNA was extracted from fungal samples ground to a fine powder in sterile centrifuge tubes using sterile rods. DNA extraction was performed with the Genomic DNA Purification Kit (Qiagen GmbH, Hilden, Germany) according to the manufacturer’s instructions, and the purified DNA was stored at -20 °C. The PCR reaction mixture (25 µL) consisted of 1 µL DNA template, 1 µL each of forward and reverse primers (10 µM), 9.5 µL ddH_2_O, and 12.5 µL 2× Taq PCR Master Mix (with dye; TIANGEN, China). The nuclear ribosomal small subunit (nrSSU) was amplified with primers NS1 and NS4 (White et al. 1990), the internal transcribed spacer (ITS) region with ITS4 and ITS5 (White et al. 1990), and the nuclear ribosomal large subunit (nrLSU) with LR0R and LR5 (Vilgalys and Hester 1990; Hopple 1994). The translation elongation factor 1-α (*tef*-*1α*) was amplified using primers 2218R and 983F (Rehner and Buckley 2005), while the largest (*rpb1*) and second largest (*rpb2*) subunits of RNA polymerase II were amplified with primer pairs RPB1/RPB1Cr_oph and fRPB2-5F/fRPB2-7cR, respectively (Liu et al. 1999; Castlebury et al. 2004; Araújo et al. 2018). All PCR amplifications were performed following the protocol described by Wang et al. (2015). PCR products were separated by electrophoresis on 1.0% agarose gels, purified using the Gel Band Purification Kit (Bio Teke Co., Ltd., Beijing, China), and sequenced on an automated sequencer (BGI Co., Ltd., Shenzhen, China). Newly generated DNA sequences have been deposited in GenBank, and accession numbers are listed in Table 1.

**Table 1.** Specimen information and GenBank accession numbers of sequences used in this study.

**Species**	**Voucher/information**	**Host/Substrate**	**GenBank accession number**						**Reference**
			**ITS**	**nrSSU**	**nrLSU**	***tef*-*1α***	***rpb1***	***rpb2***	
*Conoideocrella huteorostata*	NHJ 12516	Lepidoptera	EF468994	JN049860	EF468849	EF468800	EF468905	EF468946	Sung et al. (2007a)
*Conoideocrella tenuis*	NHJ 6293	Lepidopteran pupa	EU369112	JN049862	EU369044	EU369029	EU369068	EU369087	Sung et al. (2007a)
*Keithomyces indicus*	INFCCI5105	Soil	OL584173	OL584170	OL584176	OM032805	N/A	OM032808	Crous et al. (2022)
*Keithomyces indicus*	INFCCI5106	Soil	OL584174	NR178169	OL584177	OM032806	N/A	OM032809	Crous et al. (2022)
*Keithomyces acicularis*	JCM 33284^T^	Soil	LC435738	LC435734	LC435741	LC462188	N/A	N/A	Iwasaki et al. (2019)
*Keithomyces acicularis*	JCM 33285	Soil	LC435739	LC463198	LC435742	LC462189	N/A	N/A	Iwasaki et al. (2019)
*Keithomyces apolzaniae*	MST-F29240	Tasmania	N/A	PX788517	N/A	N/A	N/A	N/A	Tan et al. (2025)
*Keithomyces apolzaniae*	MST-F28739	Tasmania	N/A	PV554940	PV554947	PV574443	N/A	PV574442	Tan et al. (2025)
*Keithomyces carneus*	CBS 399.59	Soil	N/A	MT078887	MH869445	N/A	N/A	N/A	Sung et al. (2007b)
*Keithomyces carneus*	CBS 239.32^T^	Sand dune	EF468988	AY624171	EF468843	EF468789	EF468894	EF468938	Sung et al. (2007b)
*Keithomyces echinosporus*	CGMCC 3.25517	Soil	OR680904	OR680542	OR680609	OR858936	N/A	OR842957	Zhang et al. (2024)
*Keithomyces neogunnii*	GZUHSB13050301^T^	Lepidoptera larva	KU729721	KU729715	N/A	KU729726	KU729731	N/A	Wen et al. (2017)
*Keithomyces neogunnii*	GZUHSB13050302	Lepidoptera larva	KU729722	KU729716	N/A	KU729727	KU729732	N/A	Wen et al. (2017)
***Keithomyces ophiocordycipiticola***	**GMB 3174^T^**	***Ophiocordyceps* sp**.	**PX969544**	**PX969548**	**PX969556**	**PX959618**	**PX959610**	**PX959614**	**This study**
***Keithomyces ophiocordycipiticola***	**GMB 3175**	***Ophiocordyceps* sp**.	**PX969545**	**PX969545**	**PX969557**	**PX959619**	**PX959611**	**PX959615**	**This study**
*Marquandomyces marquandii*	CBS 182.27^T^	Soil	EF468990	AY624193	EF468845	EF468793	EF468899	EF468942	Sung et al. (2007b)
*Marquandomyces marquandii*	CBS 128893	*Hygrocybe virginea*	N/A	MH865143	MH876582	N/A	N/A	N/A	Sung et al. (2007b)
*Marquandomyces* sp.	CBS 127132	Soil	MT078872	MT078882	MT078857	MT078849	MT078865	MT078922	Sung et al. (2007b)
*Marquandomyces* sp.	CBS 129413	Soil	MT078874	MT561567	MT078859	MT078851	MT078867	N/A	Sung et al. (2007b)
*Metapochonia bulbillosa*	CBS 145.70^T^	*Picea abies*	AF339591	AJ292410	AF339542	EF468796	EF468902	EF468943	Sung et al. (2007b); Sung et al. (2001)
*Metapochonia bulbillosa*	JCM 18596	*Picea abies*	AB758252	N/A	N/A	AB758460	AB758663	AB758690	Sung et al. (2007b)
*Metapochonia goniodes*	CBS 891.72^T^	Nematoda	AF339599	AJ292409	MH872319	DQ522354	DQ522401	DQ522458	Spatafora et al. (2007)
*Metapochonia rubescens*	CBS 464.88^T^	Nematode egg	AF339615	MH862138	MH873830	EF468797	EF468903	EF468944	Sung et al. (2007b)
*Metapochonia suchlasporia*	CBS 251.83^T^	Nematode egg	N/A	MH861580	MH873311	KJ398790	KJ398601	KJ398697	Kepler et al. (2014); Vu et al. (2019)
*Metapochonia suchlasporia*	CBS 248.83^T^	Nematode egg	N/A	MH861579	MH873310	KJ398789	KJ398600	KJ398696	Kepler et al. (2014); Vu et al. (2019)
*Metarhizium album*	ARSEF 2082	Hemiptera	DQ522560	AY375446	DQ518775	DQ522352	DQ522398	DQ522452	Kepler et al. (2012a)
*Metarhizium anisopliae*	CBS 130.71^T^	*Avena sativa*	MT078868	MT078884	MT078853	MT078845	MT078861	MT078918	Gisbert (1993)
*Metarhizium brunneum*	ARSEF 2107^T^	Coleoptera	N/A	KC178691	MH868397	EU248855	EU248907	EU248935	Bischoff et al. (2009)
*Metarhizium cf. album*	ARSEF 2179	Hemiptera	N/A	N/A	N/A	KJ398807	KJ398618	KJ398716	Kepler et al. (2012a)
*Metarhizium chaiyaphumense*	BCC 19020	Hemiptera: Cicadidae	HQ165654	HQ165694	HQ165716	HQ165675	HQ165737	HQ165635	Luangsa-ard et al. (2017)
*Metarhizium chaiyaphumense*	BCC 78198^T^	Hemiptera: Cicadidae	KX369596	N/A	KX369593	KX369592	KX369594	KX369595	Luangsa-ard et al. (2017)
*Metarhizium flavoviride*	ARSEF 2025	Coleoptera	N/A	AF138269	N/A	KJ398804	KJ398614	KJ398712	Kepler et al. (2014)
*Metarhizium flavoviride*	CBS 218.56^T^	Coleoptera	N/A	MH857590	MH869139	KJ398787	KJ398598	KJ398694	Kepler et al. (2014); Vu et al. (2019)
*Metarhizium gaoligongense*	CCTCCM2016588^T^	Soil	KY087812	KY087808	KY087816	KY087820	KY087824	KY087826	Chen et al. (2018)
*Metarhizium globosum*	ARSEF 2596^T^	Lepidoptera	N/A	HQ331459	N/A	EU248846	EU248898	EU248926	Bischoff et al. (2009)
*Metarhizium guizhouense*	ARSEF 6238	Lepidoptera	N/A	N/A	N/A	EU248857	EU248909	EU248937	Bischoff et al. (2009)
*Metarhizium guizhouense*	CBS 258.90	Lepidoptera	N/A	HQ331448	N/A	EU248862	EU248914	EU248942	Bischoff et al. (2009)
*Nigelia aurantiaca*	BCC 19950	Lepidoptera	GU979934	N/A	GU979943	GU979952	GU979961	GU979967	Luangsa-ard et al. (2017)
*Nigelia aurantiaca*	BCC 37621	Lepidoptera	GU979937	N/A	GU979946	GU979955	GU979964	GU979970	Luangsa-ard et al. (2017)
*Nigelia martialis*	EFCC 6863	Lepidoptera	N/A	N/A	JF415975	JF416016	N/A	JF415995	Kepler et al. (2012b)
*Nigelia martialis*	HMAS 197472	Coleoptera: Cerambycidae	JF415955	N/A	JF415973	JF416015	JN049892	JF415994	Kepler et al. (2012b)
*Papiliomyces albostromaticus*	YHH 23070027	Hepialidae	OR770494	OR770519	OR770504	PP479838	PP203269	PP479841	Chen et al. (2024)
*Papiliomyces albostromaticus*	YFCC 23079297	Hepialidae	OR775107	OR775109	OR775108	PP479839	PP203270	PP479842	Chen et al. (2024)
*Papiliomyces albostromaticus*	YHH 2307003	Hepialidae	OR770493	OR770518	OR770503	PP479837	PP203268	PP479840	Chen et al. (2024)
***Papiliomyces huangpingensis***	**GMB 3172^T^**	** Lepidoptera **	**PX969542**	**PX969546**	**PX969554**	**PX959616**	**PX959608**	**PX959612**	**This study**
***Papiliomyces huangpingensis***	**GMB 3173**	** Lepidoptera **	**PX969543**	**PX969547**	**PX969555**	**PX959617**	**PX959609**	**PX959613**	**This study**
*Papiliomyces liangshanensis*	EFCC 1452	Lepidoptera larva	EF468962	N/A	EF468815	EF468756	N/A	N/A	Sung et al. (2007b)
*Papiliomyces liangshanensis*	EFCC 1523	Lepidoptera larva	EF468961	N/A	EF468814	EF468755	N/A	EF468918	Sung et al. (2007b)
*Papiliomyces longiclavatus*	YC 20061403^T^	Lepidoptera larva	MZ702112	MZ702080	MZ702101	MZ955880	MZ955876	MZ955872	Zhang et al. (2024)
*Papiliomyces longiclavatus*	YC 20061407	Lepidoptera larva	MZ702114	MZ702082	MZ702103	MZ955882	N/A	N/A	Zhang et al. (2024)
*Papiliomyces puniceus*	BUM 838^T^	Lepidoptera	OM951244	OM955149	OM951249	N/A	OM988194	OM988189	Chen et al. (2023)
*Papiliomyces puniceus*	BUM 1214	Lepidoptera	OM951245	OM955150	OM951250	OM988198	OM988195	OM988190	Chen et al. (2023)
*Papiliomyces shibinensis*	GZUHSB13050311	Lepidoptera	KR153588	KR153585	N/A	KR153589	KR153590	N/A	Wen et al. (2015)
“*Papiliomyces sinensis*”	DY05831^T^	Lepidoptera larva	N/A	PV082709	PV082830	PV171229	PV171123	PV171171	Chen et al. (2025b)
“*Papiliomyces sinensis*”	DY05832	Lepidoptera larva	N/A	PV082710	PV082831	PV171230	PV171124	PV171172	Chen et al. (2025b)
*Papiliomyces sinensis*	GMBC 3053^T^	*Napialus* larva	PQ636500	PQ636503	PQ636506	PQ660655	PQ660658	PQ660661	Chen et al. (2025a)
*Papiliomyces sinensis*	GMBC 3054	*Napialus* larva	PQ636501	PQ636504	PQ636507	PQ660656	PQ660659	PQ660662	Chen et al. (2025a)
*Pochonia boninensis*	JCM 18597	Soil	AB758255	AB709858	AB709831	AB758463	AB758666	AB758693	Nonaka et al. (2013)
*Pochonia chlamydosporia*	CBS 504.66^T^	Soil	AF339593	AJ292398	AF339544	EF469069	EF469098	EF469120	Sung et al. (2007a); Sung et al. (2001)
*Pochonia chlamydosporia*	CBS 101244	Mollusca	DQ522544	JN049821	DQ518758	DQ522327	DQ522372	DQ522424	Spatafora et al. (2007)
*Pochonia globispora*	CBS 203.86^T^	Soil	N/A	DQ516079	MH873631	N/A	N/A	N/A	Zare and Gams (2007)
*Purpureomyces khaoyaiensis*	BCC 14290	Lepidoptera larva	KX983469	JN049869	KX983463	KX983458	N/A	KX983466	Hywel-Jones (1994)
*Purpureomyces khaoyaiensis*	BCC 44287	Lepidoptera larva	KX983470	N/A	KX983464	KX983459	N/A	KX983467	Hywel-Jones (1994)
*Purpureomyces pyriformis*	BCC 85074^T^	Lepidoptera larva	N/A	MN781929	MN781873	MN781730	MN781775	MN781821	Mongkolsamrit et al. (2020)
*Purpureomyces pyriformis*	BCC 85348	Lepidoptera larva	N/A	MN781927	MN781871	MN781728	MN781773	MN781820	Mongkolsamrit et al. (2020)
*Rotiferophthora angustispora*	CBS 101437	Bdelloid rotifers	AF339584	N/A	AF339535	AF543776	DQ522402	DQ522460	Kawaliło et al. (2025)
*Samsoniella asiatica*	YFCC 869^T^	Lepidoptera pupa	OQ476497	OQ476473	OQ476505	OQ506153	OQ506195	OQ506187	Wang et al. (2023)
*Samsoniella hepiali*	YFCC 868	Hepialidae pupa	OQ476502	OQ476484	OQ476510	OQ506158	OQ506200	OQ506192	Wang et al. (2023)
*Sungia yongmunensis*	EFCC 2131^T^	Lepidoptera	EF468977	JN049856	EF468833	EF468770	EF468876	KJ398690	Sung et al. (2007a)
*Sungia yongmunensis*	EFCC 2135	Lepidoptera	EF468979	N/A	EF468834	EF468769	EF468877	N/A	Sung et al. (2007a)
*Yosiokobayasia kusanagiensis*	TNS F18494	Lepidoptera	JF415954	JN049873	JF415972	JF416014	JN049890	N/A	Kepler et al. (2012a)
*Yosiokobayasia kusanagiensis*	BUM 1307	Lepidoptera	OM951246	OM955151	OM951251	OM988199	N/A	OM988191	Sung et al. (2007b)

**Boldface**: data generated in this study; **T**: ex-type material. Institutional acronyms: **ARSEF**: Agricultural Research Service Collection of Entomopathogenic Fungal Cultures (culture collection); **BCC**: BIOTEC Culture Collection (culture collection); **CBS**: Westerdijk Fungal Biodiversity Institute (culture collection); **CGMCC**: China General Microbiological Culture Collection Center; **GMB**: Herbarium of Guizhou Medical University (herbarium); **GMBC**: Guizhou Medical University Culture Collection (culture collection); **GZUHSB**: Herbarium of Guizhou University; **JCM**: Japan Collection of Microorganisms; **NHJ**: National Herbarium of Japan (herbarium); **TNS**: National Museum of Nature and Science (herbarium).

### Phylogenetic analyses

Taxa included in the phylogenetic analyses were selected to represent the major evolutionary lineages of the genera *Papiliomyces*, *Keithomyces*, and their closely related genera within Clavicipitaceae, with an emphasis on species for which sequence data from multiple loci were available. Sequence data for six loci (nrSSU, ITS, nrLSU, *tef*-*1α*, *rpb1*, and *rpb2*) were obtained from GenBank. Relevant taxonomic information and accession numbers are provided in Table 1. Sequence alignments were generated using MAFFT v.7 (https://mafft.cbrc.jp/alignment/server/, accessed on 7 January 2026) and MEGA v.7.0.26 (Kumar et al. 2016), with manual adjustments performed where necessary. Individual gene alignments were subsequently concatenated into a combined dataset using MEGA v.7.0.26. Phylogenetic relationships were inferred using Maximum Likelihood (ML) and Bayesian Inference (BI) methods. ML analyses were performed using both RAxML v.7.0.3 and IQ-TREE v.2.1.3 (Edler et al. 2021). For IQ-TREE analyses, the GTR+F+I+G4 model was selected as the best-fit substitution model, and branch support was assessed using ultrafast bootstrapping (Minh et al. 2020). For RAxML analyses, branch support was evaluated based on 1,000 rapid bootstrap replicates. For BI analyses, the best-fit substitution models were selected using jModelTest v.2.1.4 (Darriba et al. 2012). The GTR+I+G model was applied to the nrSSU, ITS, nrLSU, and *tef*-*1α* partitions, while the GTR+I model was used for the *rpb1* and *rpb2* partitions. Bayesian inference was conducted in MrBayes v.3.2.7a (Ronquist et al. 2012) with 5 million generations. *Samsoniella asiatica* YFCC 869 and *Samsoniella hepiali* YFCC 868 were designated as outgroup taxa. The resulting phylogenetic trees were visualized and edited using FigTree v.1.4.4 and iTOL v.7 (Letunic and Bork 2024).

## Results

### Phylogenetic analyses

**Figure 1.**
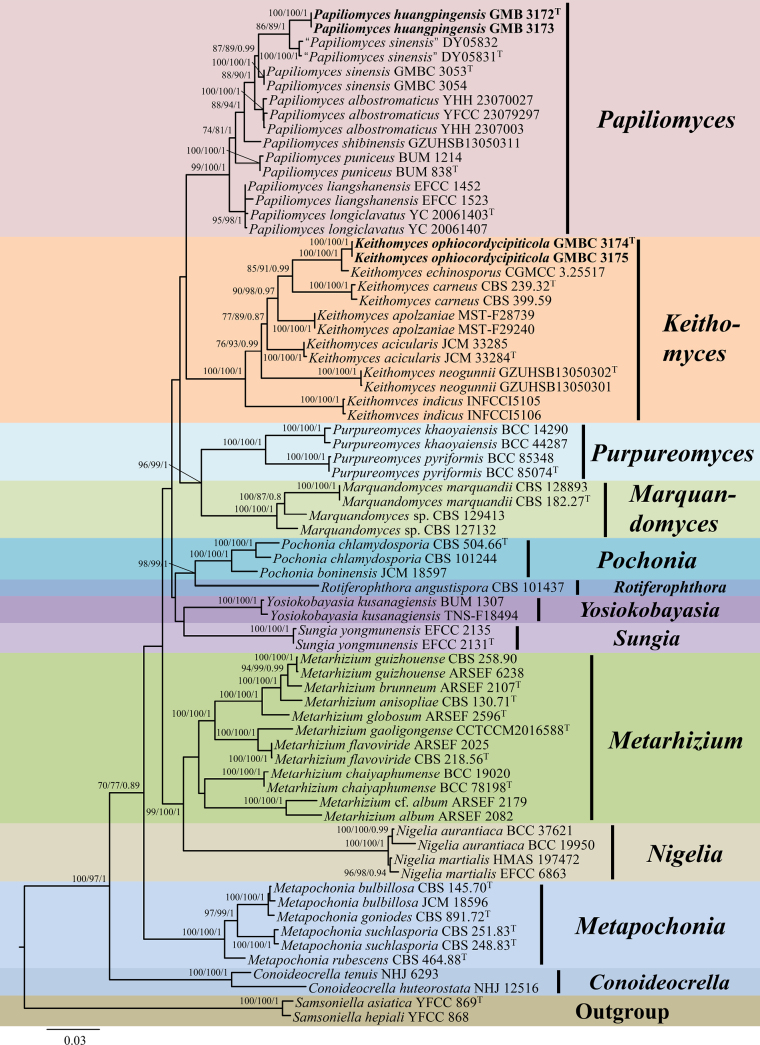
Phylogenetic tree of *Keithomyces*, *Papiliomyces*, and related genera based on a combined six-locus dataset (nrSSU, ITS, nrLSU, *tef*-*1α*, *rpb1*, and *rpb2*). Branch support values from RAxML, IQ-TREE, and Bayesian Inference (RAxML-BS/IQ-TREE-BS/BI-PP) greater than 70%/70%/0.80 are shown. Ex-type materials are indicated with “T”. Sequences generated in this study are shown in bold.

Phylogenetic relationships among the two newly identified species of *Papiliomyces* and *Keithomyces*, together with their closely related genera, were inferred based on a six-locus dataset comprising nrSSU, ITS, nrLSU, *tef*-*1α*, *rpb1*, and *rpb2*. The concatenated alignment had a total length of 5,598 bp, including 1,108 bp of nrSSU, 752 bp of ITS, 912 bp of nrLSU, 987 bp of *tef*-*1α*, 768 bp of *rpb1*, and 1,071 bp of *rpb2*. The dataset comprised 71 taxa representing Clavicipitaceae, with *Samsoniella asiatica* YFCC 869 and *Samsoniella hepiali* YFCC 868 selected as outgroup taxa. Phylogenetic analyses were conducted using Maximum Likelihood (ML) methods implemented in RAxML and IQ-TREE, as well as Bayesian Inference (BI). All three analytical approaches yielded highly congruent tree topologies. Fig. 1 summarizes the phylogenetic relationships among the 71 clavicipitaceous taxa and illustrates the placement of the focal lineages.

Phylogenetic analyses supported the monophyly of the genera *Papiliomyces* (99%/100%/1) and *Keithomyces* (100%/100%/1), which were resolved as sister genera. The two species described in this study were clustered within *Papiliomyces* and *Keithomyces*, respectively, and formed distinct lineages separate from previously known species. Within *Papiliomyces*, *P. huangpingensis* sp. nov. formed a sister relationship with “*Papiliomyces sinensis*” (DY05831 and DY05832) reported by Chen et al. (2025b), with strong support (86%/89%/1). These two strains were clearly separated from *Papiliomyces sinensis*Chen et al. (2025a) (the legitimate name with nomenclatural priority), suggesting that they represent a distinct undescribed taxonomic entity.

*Keithomyces ophiocordycipiticola* sp. nov. formed a distinct lineage within *Keithomyces* and clustered together with *K. echinosporus* and *K. carneus*, with relatively high statistical support (85%/91%/0.99). The new species was clearly separated from other known *Keithomyces* taxa, including *K. apolzaniae*, *K. acicularis*, *K. neogunnii*, and *K. indicus*. In addition, the phylogenetic tree demonstrated clear generic boundaries among *Papiliomyces*, *Keithomyces*, *Purpureomyces*, *Marquandomyces*, *Pochonia*, *Yosiokobayasia*, *Sungia*, *Metarhizium*, *Nigelia*, *Metapochonia*, and *Conoideocrella*, all of which were recovered as independent monophyletic clades with moderate to high support values. Overall, these phylogenetic results support the recognition of *Papiliomyces huangpingensis* and *Keithomyces ophiocordycipiticola* as two novel species within Clavicipitaceae and contribute to a clearer understanding of the phylogenetic relationships among related genera in the family.

## Taxonomy

### Papiliomyces huangpingensis

Taxon classificationFungiSordariomycetesClavicipitaceae

H. Chen & D.X. Tang sp. nov.

05A05FC1-7FC9-5872-A6F3-F6A93D0CA88C

862700

Fig. 2

#### Etymology.

The epithet referred to the locality (huangping) where the holotype was collected.

**Figure 2.**
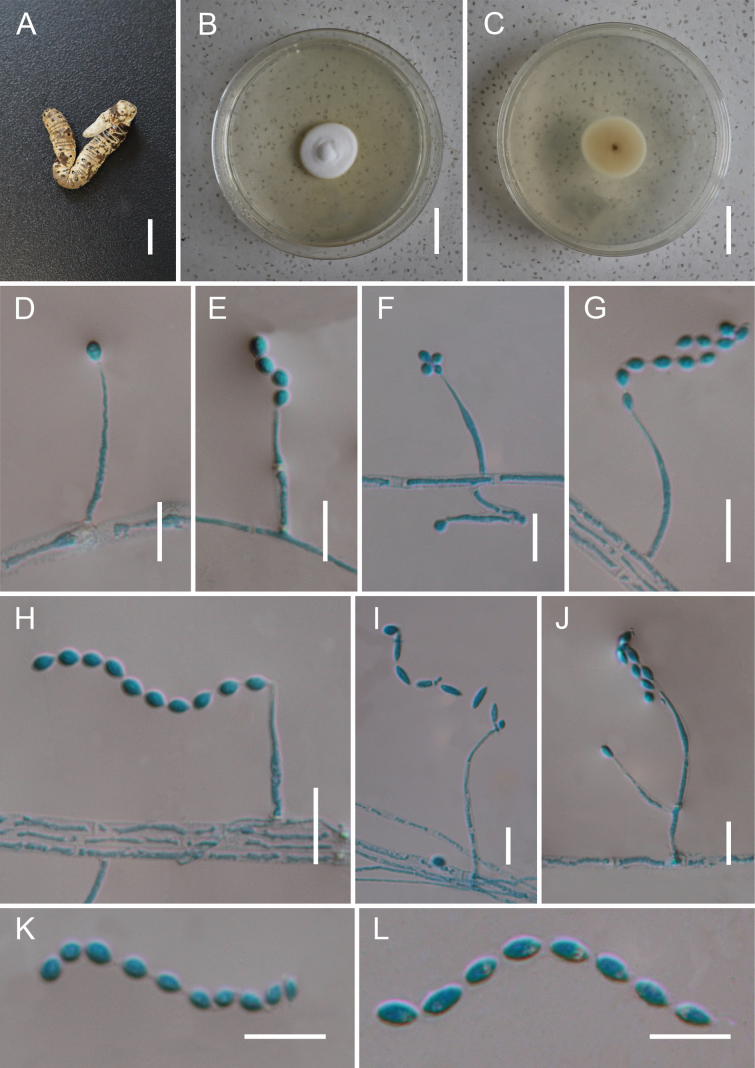
The morphological and micromorphological characteristics of *P. huangpingensis*. **A**. Morphology of *P. huangpingensis*; **B, C**. Culture character on PDA medium; **D–J**. Conidiophores, phialides, and conidia; **K, L**. Conidia. Scale bars: 1 cm (**A**); 2 cm (**B, C**); 10 μm (**D–L**).

#### Type.

China, • Guizhou Province, Qiannan Buyi and Miao Autonomous Prefecture, Huangping County (27°30'N, 108°30'E, 877 m above sea level), from larvae of Hepialidae, Mar. 2025, De-Xiang Tang (holotype: GMB 3172; ex-holotype living culture: GMBC 3172).

#### Description.

***Stromata*** solitary, fleshy, clavate, short and stout, gray to earthy yellow, arising from the head of the host, measuring 12.3–22.7 × 2.6–4.1 mm (x̄ = 15.5 × 3.5 mm, n = 5). Perithecia, asci, and ascospores not observed. Hyphae hyaline, septate, branched, smooth-walled, 0.8–2.5 µm wide. ***Conidiophores*** smooth-walled, cylindrical, mononematous, erect, aseptate, measuring 15.1–51.8 × 0.8–2.7 µm (x̄ = 28.5 × 1.7 µm, n = 30). ***Phialides*** verticillate, in whorls of two to three, usually solitary on hyphae; basal portion cylindrical to narrowly lageniform, gradually tapering toward the apex, 11.5–25 × 0.7–3.3 µm (x̄ = 16.5 × 2.5 µm, n = 30). ***Conidia*** are produced in long chains, dimorphic. *α*-conidia globose to ellipsoidal, one-celled, 2.8–4.3 × 1.9–3.8 µm (x̄ = 3.5 × 2.8 µm, n = 50); β-conidia ovoid, one-celled, 4.5–5.9 × 2.1–2.8 µm (x̄ = 4.9 × 2.5 µm, n = 50).

#### Asexual morph.

*Paecilomyces*-like.

#### Colonies.

The colony grows fast on PDA, reaching 23–24 mm in diameter after 7 days at 25 °C, surface white to gray, cottony, with increased and raised mycelial density at the center and several concentric rings formed at the margin; reverse pale yellow to brown. Hyphae hyaline, septate, branched, smooth-walled, 0.8–2.5 µm wide.

#### Host.

Larvae of Hepialidae (Lepidoptera).

#### Material examined.

China, • Guizhou Province, Qiannan Buyi and Miao Autonomous Prefecture, Huangping County (27°23'N, 108°31'E, 927 m above sea level), from larvae of Hepialidae, Mar. 2025, De-Xiang Tang (GMB 3173; living culture: GMBC 3173).

#### Notes.

*Papiliomyces huangpingensis* is resolved as sister to “*P. sinensis*” in the phylogenetic analyses but is clearly distinguished from the latter by forming an independent lineage within this clade (Fig. 1; 86%/89%/1). Morphologically, *P. huangpingensis* is most similar to *P. albostromaticus* and “*P. sinensis*”, as all three species possess fleshy, clavate, gray stromata and a *Paecilomyces*-like asexual conidiogenous structure. However, *P. huangpingensis* can be readily distinguished from both species by its markedly shorter and stouter stromata (12.3–22.7 × 2.6–4.1 mm vs. 37.0–85.7 × 2.5–3.5 mm) and by the presence of two distinct conidial morphotypes (Chen et al. 2024, 2025b).

### Keithomyces ophiocordycipiticola

Taxon classificationFungiSordariomycetesClavicipitaceae

D.X. Tang, H. Chen & O Huang sp. nov.

B4E45E13-E9AA-58BE-A0BF-737D7D6ABF03

862699

Fig. 3

#### Etymology.

The fungus was obtained as an isolate from the sclerotium of *Ophiocordyceps* sp.

**Figure 3.**
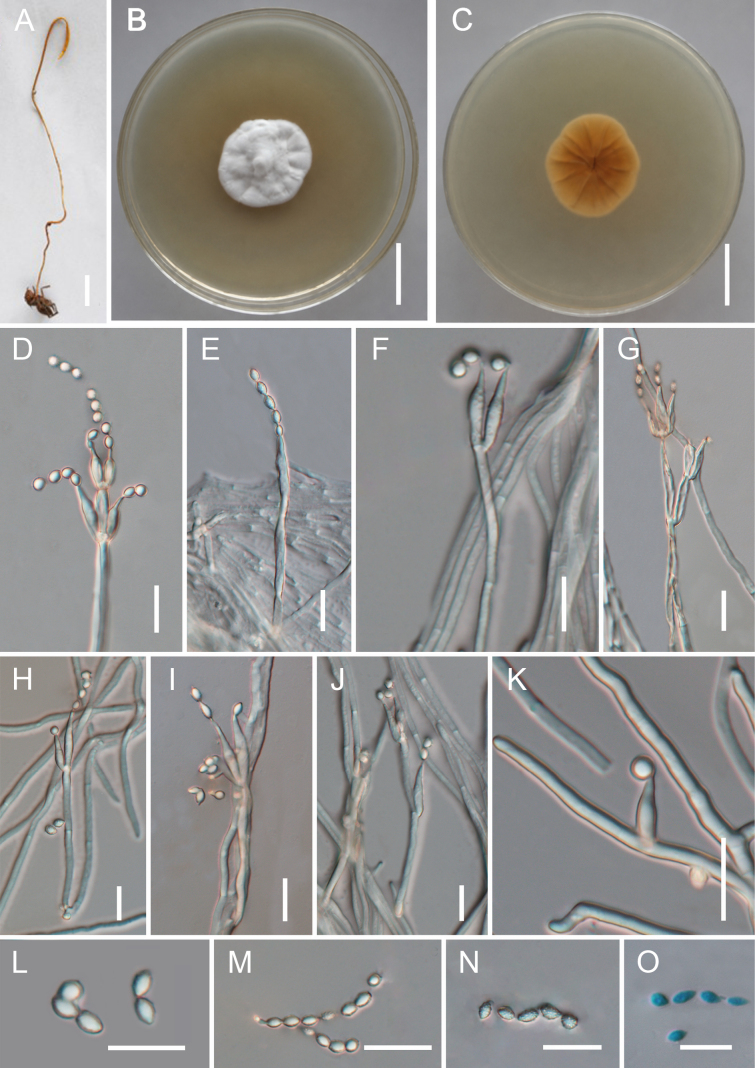
Morphology of *K. ophiocordycipiticola*. **A**. Field-collected *Ophiocordyceps* sp. specimen, from which *K. ophiocordycipiticola* (strain GMBC 3174) was isolated; **B, C**. Culture characteristics on potato dextrose agar; **D–K**. Conidiophores, phialides, and conidia; **L–O**. Conidia. Scale bars: 1 cm (**A**); 2 cm (**B, C**); 10 μm (**D–O**).

#### Type.

China, • Yunnan Province, Puer City, Lancang Lahu Autonomous County (22°54'N, 100°30'E, 1277 m above sea level), from the sclerotium of *Ophiocordyceps* sp., Sept. 2025, De-Xiang Tang (holotype: GMB 3174; ex-holotype living culture: GMBC 3174).

#### Description.

Stroma, perithecia, asci, and ascospores not observed. Mycelium solitary or arranged in parallel hyphal bundles, branched, septate, pigmented or hyaline, thin- and smooth-walled, 1.9–3.1 µm wide. ***Conidiophores*** mononematous, unbranched, arising laterally from superficial hyphae, smooth-walled, hyaline, simple or verticillate, 30.5–75 × 1.5–3.4 µm (x̄ = 46 × 2.5 µm, n = 30). ***Phialides*** solitary, sometimes produced directly from superficial hyphae, or in groups of 2–5, produced terminally on conidiophores; acerose with an elongated neck, neck straight to slightly tapered towards the apex, base narrow, smooth-walled, hyaline, 2–24 × 1.8–3.5 µm (x̄ = 18 × 2.8 µm, n = 30). ***Conidia*** subglobose to globose, occasionally fusiform, catenate, echinulate, hyaline, 2–5.5 × 2–2.8 µm (x̄ = 3.5 × 2.2 µm, n = 30). Chlamydospores absent.

#### Asexual morph.

*Paecilomyces*-like.

#### Colonies.

Colonies fast-growing on PDA, reaching 27–29 mm in diameter after 7 days at 25 °C, surface white to gray, rugose, with a firm texture, mycelial density increased and raised at the center, reverse pale yellow to brown. Hyphae hyaline, septate, branched, smooth-walled, 1.9–3.1 µm wide.

#### Host.

*Ophiocordyceps* sp.

#### Material examined.

China, • Yunnan Province, Puer City, Lancang Lahu Autonomous County (22°44'N, 100°26'E, 1257 m above sea level), from the sclerotium of *Ophiocordyceps* sp., Sept. 2025, De-Xiang Tang (living culture: GMBC 3175).

#### Notes.

In the phylogenetic analyses, *K. ophiocordycipiticola* clustered with *K. echinosporus*, forming a strongly supported clade in which *K. ophiocordycipiticola* was recovered as sister to *K. echinosporus* (Fig. 1; 100%/100%/1). Morphologically, the two species are similar in possessing mononematous, unbranched conidiophores, acerose phialides, and hyaline echinulate conidia. However, *K. ophiocordycipiticola* can still be distinguished from *K. echinosporus* by several stable morphological characteristics. Compared with *K. echinosporus*, *K. ophiocordycipiticola* possesses generally shorter and narrower conidiophores (30.5–75 × 1.5–3.4 µm vs. 40–95 × 2.5–3.5 µm; Zhang et al. 2024). In addition, the hyphae of *K. ophiocordycipiticola* are sometimes arranged in parallel hyphal bundles, a feature that has not been reported in *K. echinosporus*. The conidial morphology also differs slightly between the two species. Conidia of *K. ophiocordycipiticola* are predominantly subglobose to globose and only occasionally fusiform, whereas those of *K. echinosporus* are more frequently ellipsoidal or fusiform. These morphological differences, together with the phylogenetic evidence, support the recognition of *K. ophiocordycipiticola* as a distinct species within *Keithomyces*.

Furthermore, *K. ophiocordycipiticola* was isolated from the sclerotium of an *Ophiocordyceps* sp., representing an unusual ecological occurrence within the genus and suggesting that the ecological diversity of *Keithomyces* may be broader than previously recognized.

## Discussion

This study, based on integrated multi-gene phylogenetic analyses and detailed morphological observations, systematically investigated the taxonomic positions of *P. huangpingensis* and *K. ophiocordycipiticola*, two newly discovered species from China. Phylogenetic analyses demonstrated that both taxa represent independent evolutionary lineages within their respective genera, each supported by strong statistical values and consistent morphological distinctions, thereby justifying their recognition as new species.

*Papiliomyces huangpingensis* was resolved as a sister to “*P. sinensis”* but formed an independent lineage within the *Papiliomyces* clade. Morphologically, *P. huangpingensis* closely resembles “*P. sinensis”* and *P. albostromaticus* in sharing fleshy, clavate, gray stromata and a *Paecilomyces*-like asexual morph. However, it can be clearly distinguished from these taxa by its markedly shorter and stouter stromata and by the presence of two distinct conidial morphotypes. These stable morphological differences, in combination with its distinct phylogenetic placement, support the delimitation of *P. huangpingensis* as a separate species within *Papiliomyces*. *Keithomyces ophiocordycipiticola* formed a strongly supported sister relationship with *K. echinosporus* in the phylogenetic analyses. Morphologically, these species are similar in possessing mononematous conidiophores, acerose phialides, and hyaline, echinulate conidia. Nevertheless, *K. ophiocordycipiticola* can be readily distinguished from *K. echinosporus* by its significantly shorter conidiophores. The congruence between molecular data and these diagnostic morphological characters confirms the taxonomic independence of *K. ophiocordycipiticola*.

In addition to morphological and phylogenetic evidence, the ecological origin of *K. ophiocordycipiticola* is noteworthy. Unlike other known species of the genus, which have predominantly been isolated from soil or soil-associated substrates (Mongkolsamrit et al. 2020; Zhang et al. 2024), *K. ophiocordycipiticola* was isolated from the sclerotia of *Ophiocordyceps* sp. Although its ecological role remains unclear, this finding expands the known ecological occurrence of *Keithomyces*. A similar case was reported by Shen et al. (2026), who described *Leptobacillium hepiali* isolated from the sclerotium of *Ophiocordyceps* species, suggesting that the sclerotia of entomopathogenic fungi may harbor previously overlooked clavicipitaceous fungi.

Taken together, the discovery of *P. huangpingensis* and *K. ophiocordycipiticola* highlights the importance of integrative taxonomy combining morphology, phylogeny, and ecological data. While traditional morphological characters remain indispensable for species diagnosis, molecular divergence and ecological observations often reveal hidden diversity that is not apparent from morphology alone. Continued exploration of non-traditional substrates, such as stromata and sclerotia of entomopathogenic fungi, is therefore expected to uncover additional cryptic diversity and contribute to a more natural and stable classification within Clavicipitaceae.

### Nomenclatural note

The binomial *Papiliomyces sinensis* was first validly published by Chen et al. (2025a) on 15 May 2025 in MycoKeys, based on the ex-type strain GMBC 3053 collected from Guizhou Province, China. Five-and-a-half months later, Chen et al. (2025b) published the identical binomial for a distinct taxon on 30 October 2025 in Mycosphere, which constitutes a later homonym and is therefore illegitimate under Article 53.1 of the International Code of Nomenclature for algae, fungi, and plants (ICN). Our multilocus phylogenetic analyses confirmed that strains DY05831 and DY05832 (assigned to “*P. sinensis*” by Chen et al. (2025b)) form a well-supported monophyletic clade sister to *P. huangpingensis* sp. nov., and are clearly phylogenetically distinct from the authentic *P. sinensis*Chen et al. (2025a). We therefore treat these isolates as “*Papiliomyces sinensis*” Chen et al. (2025b) and recommend that this taxon be formally renamed with a new epithet in a subsequent publication.

## Supplementary Material

XML Treatment for Papiliomyces huangpingensis

XML Treatment for Keithomyces ophiocordycipiticola

## Additional information

### Artificial Intelligence (AI) use

The authors accept full responsibility for the content of the manuscript, including the disclosure of any use of AI.

No AI tools were used in the preparation of this manuscript.

### Author ORCIDs

H. Chen https://orcid.org/0009-0008-0291-3571

Q.-R. Li https://orcid.org/0000-0001-8735-2890

Y. Wang https://orcid.org/0000-0002-1262-6700

X. Sheng https://orcid.org/0000-0002-4333-9106

### Data availability

All of the data that support the findings of this study are available in the main text.
